# Sex-specific percentiles for bodyweight and height in children born with esophageal atresia: a registry-based analysis 2001–2021

**DOI:** 10.1186/s12887-023-03842-4

**Published:** 2023-01-18

**Authors:** Tatjana Tamara König, Maria-Christina Stefanescu, Melanie Wildermuth, Luisa Maria Frankenbach, Oliver J. Muensterer, Emilio Gianicolo

**Affiliations:** 1 Department of Pediatric Surgery, Universitätsmedizin, Johannes Gutenberg-University, 55131 Mainz, Germany; 2 Kinder und Erwachsene mit kranker Speiseröhre, KEKS e.V., Stuttgart, Germany; 3 Department of Pediatric Surgery, Dr. von Haunersches Kinderspital, Ludwig Maximilian-University Munich, Munich, Germany; 4 Institute of Medical Biostatistics, Epidemiology and Informatics (IMBEI), Universitätsmedizin, Johannes Gutenberg-University, Mainz, Germany; 5 Institute of Clinical Physiology, National Research Council, Lecce, Italy

**Keywords:** Percentiles, Esophageal atresia, Body weight, Height, Growth

## Abstract

**Background:**

Esophageal atresia (EA) is a rare malformation with a wide range of co-morbidity and associated malformations impairing weight gain and growth. The aim of this study was to calculate specific percentiles for body weight and height for children born with esophageal atresia according to sex from birth to the age of 6 years, accounting for prematurity and presence of congenital heart disease (CHD).

**Methods:**

Data was extracted from an anonymized voluntary national registry for patients born with esophageal atresia between 2001 and 2021, from birth until the age of six years. Missing values were imputed using a multiple imputation approach. In premature infants, chronological age was corrected for gestational week until the age of one year. The impact of sex and additional congenital heart disease on weight gain and growth was analysed using quartile regression models.

**Results:**

In total, 1812 examinations of 485 patients were considered and 1232 examinations of 301 patients were finally included. Most data was available for children at birth and during the first year of life. Body weight was imputed for 3.3% and height for 12.5% of examinations. The mean body weight-for-age and length-for-age at birth according to gestational age was lower in EA patients and median body weight developed along the tenth percentile compared to the general population. Median height-for-age was at the 50th percentile during the first months of life, before crossing to lower percentiles before the age of one year. CHD had an additional negative impact on growth and weight gain, especially during the first year of life.

**Conclusions:**

Children with EA had a reduced bodyweight and -height compared to the general population. Therefore, specific percentile curves are helpful to evaluate growth and development. Especially during the first year of life, particular attention is necessary and complications leading to a reduced calorie intake should be treated without delay to promote timely development and growth. Cardiac co-morbidities may further compromise weight gain in these patients, implying that such patients should be under even closer surveillance.

**Supplementary Information:**

The online version contains supplementary material available at 10.1186/s12887-023-03842-4.

## Introduction

Esophageal atresia (EA) is a rare malformation with a mean incidence of 162 per year in Germany [1]. In spite of medical and surgical progress, many affected children suffer from life-long gastrointestinal problems such as relevant gastroesophageal reflux (GER) leading to esophagitis, esophageal motility problems, dysphagia, necessity for tube feeding, and failure to thrive. Up to 40% of patients are affected by respiratory symptoms [2]. Feeding difficulties are multifactorial, including esophageal, oropharyngeal and behavioural disorders [3]. Furthermore, esophageal atresia is associated with other congenital malformations in over half of all cases [4, 5], such as cardiac abnormalities (28 to 42% [4, 5]), which may influence growth and development [2]. Low birth weight is also frequently associated: 17 % of children with EA are born small for gestational age [5] and around 40% of children are born preterm [5, 6]. A series of 287 patients reports a birthweight below 1500 g in 14% of newborns with EA [4].

While a reduced body weight and height was described in many studies [6–9], factors of comorbidity and prematurity have not been adequately considered [2]. A recent study of the National French EA Registry including more than 700 patients identified prematurity (odds-ratio 2.4), low birth weight (small for gestational age, odds-ratio 2) and other co-morbidity as risk factors for undernutrition or stunting at the age of one year, independent of the type of EA or technique of repair [5]. Bodyweight at discharge was shown to be predictive of bodyweight-for-age at the ages of one and three years. The average weight-for-age score might be close to the general population by the age of three years [10]. Another German series reports a body weight below the 25th percentile in 67% of six-year-olds and 41% of patients at the age of ten years [11]. At the age of 12 years, the average height-for-age was − 0.2 (standard deviation 0.13). Ultimately, only 7% of patients remain below the 5th percentile for body weight and height in long-term follow-up [12]. In this case series with 371 patients by Deurloo et al., prematurity and GER were identified as risk factors for low weight and height [12].

Current standard reference percentile curves for infants and children 0 through 18 years in Germany were published in 2001 by Kromeyer-Hausschild et al. and are based on a systematic review of 17 regional studies [13]. In 2013, new percentiles based on the KiGGS cohort longitudinal study on the health of children, adolescents and young adults were published [14], that are very similar to Kromeyer-Hauschild, but provide more detailed data for infants and children below the age of two years [14]. For newborns, percentiles according to gestational week were published in 2006 by Voigt et al. [15].

Unfortunately, there is no national registry for children with EA in Germany. However, in 2012, the national patient organization for EA (Kinder und Erwachsene mit kranker Speiseröhre, KEKS e.V.) established a structured follow-up plan for their members, which is widely utilized and accepted in the pediatric surgical community. Families and healthcare-providers are thereby encouraged to transmit follow-up data to an anonymized database.

The aim of this study was to calculate specific percentiles for body weight and height for children born with esophageal atresia according to sex from birth to the age of 6 years, accounting for prematurity and presence of congenital heart disease.

## Methods

### Data source

Anonymized data from a voluntary registry for patients born with esophageal atresia collected in the years 2012 through 2021 by KEKS e.V. was analysed. In this paper-based registry, that provides a standardised follow-up plan for patients, standardised forms are completed by the attending physician and forwarded to the patient organisation, where they are digitalised in an anonymous data base.

### Data extraction

Patients born between November 15, 2001 and November 15, 2021, were included from birth to 72 months of age. For preterm children, chronological age at the time of the examination was corrected for gestational week during the first year of life. Examinations with missing information on sex or both bodyweight and -height were excluded.

### Imputation of data and statistical analysis

For examinations, missing information on either weight or height, data was imputed. Assuming data was missing at random, we used multiple imputation and fully conditional specification methods to retrieve missing information [16, 17]. For imputation purposes, we used information on age and accounted for sex. For each combination of sex and age group, 100 data sets were generated in order to represent a convenient random sample of missing values. Finally, since data of four examinations was available for each patient on average, we used a multilevel approach to account for the clustered nature of our data [18]. For statistical analysis, we grouped patients according to chronological age corrected for gestational age: at birthdate, at monthly intervals for patients aged 1 to 12 months, quarterly intervals for patients aged 13 through 24 months, and intervals of six months for patients aged 25 through 72 months of age at the index examination.

### Evaluation compared to the general population

Data was compared to percentiles based on the KiGGS cohort longitudinal study on the health of children, adolescents and young adults [14]. For birth weight and birth length, z-scores were calculated according to percentiles for newborns by Voigt et al. [15]. Small for gestational age was defined as below the 10th percentile or z-score below − 1.28 for sex and gestational week. Means and 95%-confidence intervals (95%-CI) were calculated.

### Evaluation of influencing factors

Sex differences and the effect of congenital heart disease were evaluated using quantile regression models [19]. Quantile regression parameters were estimated for the following percentiles: 10th, 25th, 50th, 75th, and 90th. The resampling method, which uses bootstrap procedures, was used to calculate 95%-CI.

Statistical analysis was performed using SAS software Version 9.4 (SAS Institute, Inc., Cary, NC, USA). For multiple imputation Blimp-Studio was used [20].

## Results

**Fig. 1:**
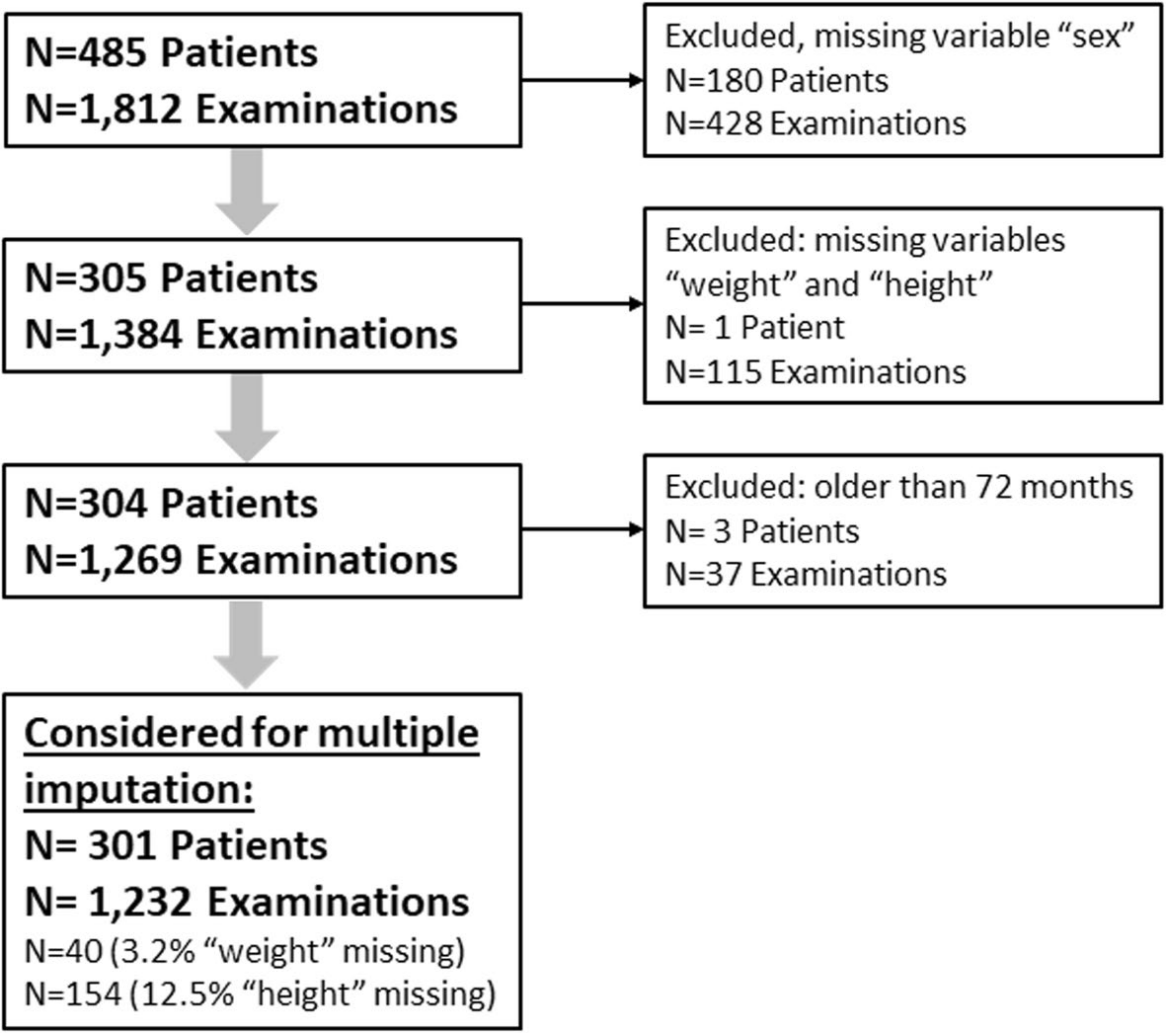
Inclusion Flowchart

In total, 1812 examinations of 485 patients born with EA during the study period of ten years were considered. Most data was available at birth (34% of examinations for weight and 36% of examinations for length) and the first year of life (42% of examinations for weight and 39% of examinations for length). Due to missing data on either sex, age older than 72 months or missing information on both bodyweight and height, a total of 184 patients and 580 single examinations were excluded (Fig. 1). Finally, 1232 examinations of 301 patients were included. The variable bodyweight was imputed in 40 (3.3%) examinations, and height in 154 (12.5%) examinations (Fig. 1). More detailed information on long-term medical history is provided in Table 1.

**Table 1:** Patient characteristics

		***n*** (%)
**Gross type**	A	16 (5.3%)
	B	6 (2.0%)
	C	264 (87.7%)
	D	8 (2.7%)
	E	5 (1.7%)
	other	2 (0.7%)
**Surgical Repair**	primary esophageal anastomosis and repair of a tracheoesophageal fistula	244 (81.1%)
	primary repair of a tracheoesophageal fistula and delayed esophageal anastomosis	7 (2.3%)
	primary repair of a tracheoesophageal fistula only	9 (3.0%)
	delayed esophageal anastomosis only	16 (5.3%)
	other	25 (8.3%)
**Congenital heart disease**		104 (34. 6%)
	not specified	25 (8.3%)
**Genetic syndrome**		8 (2.7%)
**Gestational age**	preterm	98 (32.6%)
	term	184 (61.1%)
	not specified	19 (6.3%)

### Gestational age, birth weight and -length

Data on gestational age, bodyweight and -length at birth were available for 276 patients (118 female, 158 male). The information on length was missing for 28 newborns. In total, 98 children (32.6%) were born premature and 112 (40.7%) small for gestational age. The mean gestational age was 37.1 weeks (95%-CI: 36.7–37.6, range 26–42). The mean body weight-for-age at birth was − 1.0 (95%-CI: − 1.1; − 0.9) and length-for-age − 0.6 (95%-CI: − 0.7; − 0.4) according to gestational age [15]. There was no difference in gestational age between sexes (male 37.1 weeks, 95%-CI: 36.5–37.7, female 37.2 weeks, 95%-CI: 36.5–37.8). Males had a slightly lower mean z-score for birth weight (male − 1.1, 95%-CI: − 1.2; − 0.9, female − 1.0, 95%-CI: − 1.1; − 0.8) and length (male − 0.6, 95%-CI: − 0.8; − 0.4, female − 0.5, 95%-CI: − 0.7; − 0.2).

### Percentiles

**Fig. 2:**
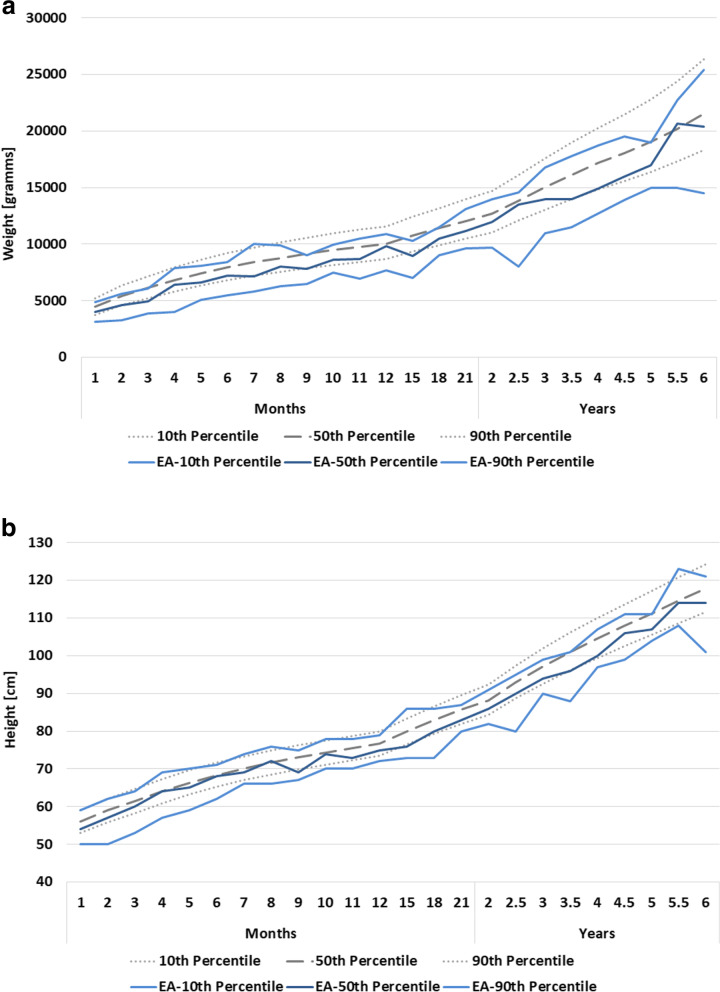
Percentiles for body weight (**a**) and height (**b**) for males with esophageal atresia (EA) during the first 6 years of life compared to the general male population [14]. Germany, 2001–2021. Source: patient registry of KEKS e.V.

**Fig. 3:**
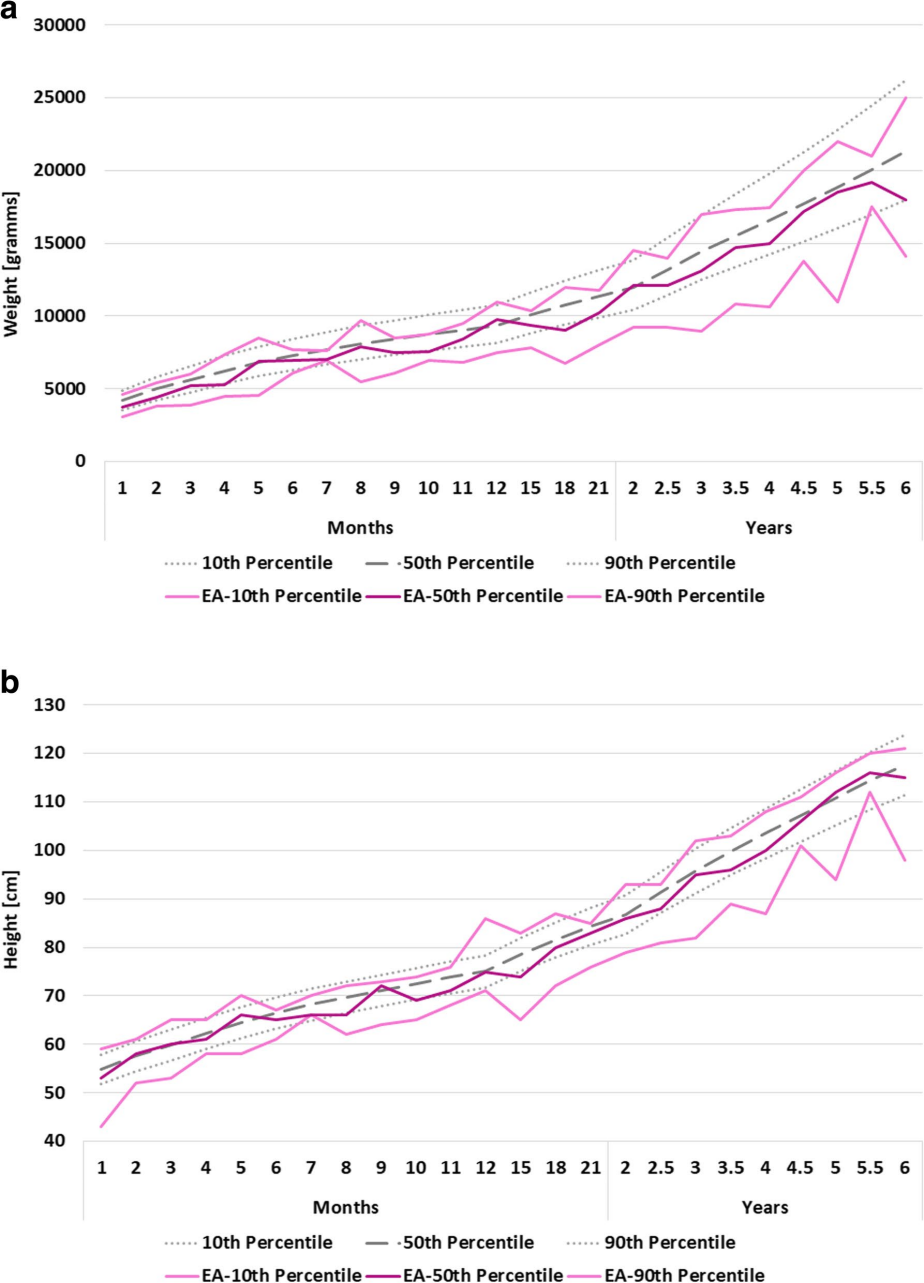
Percentiles for body weight (**a**) and height (**b**) for females with esophageal atresia (EA) during the first 6 years of life compared to the general male population [14]. Germany, 2001–2021. Source: patient registry of KEKS e.V.

Percentiles for bodyweight and –height for EA patients according to gender are presented in Tables 2 and 3, respectively. Children born with EA had a lower median for bodyweight and length compared to the general population for the most part (Figs. 2a, b and 3a, b). In both males and females, median bodyweight developed along the 10th percentile of the general population (Fig. 2a, 3a). The median height developed along the median of the general population during the first six months of life, before crossing percentiles to develop along the 10th of the general population around the age of 12 months (Fig. 2b, 3b). All curves of EA patients show a certain fluctuation due statistical uncertainty that must be considered while interpreting the data.

**Table 2:** Percentiles for bodyweight and –height in males with esophageal atresia according to age (months) after multiple imputation. Germany, 2001–2021. Source: patient registry of KEKS e.V.

	*Weight (gramms)*							*Height (cm)*						
*Age (months)*	*EA-P5*	*EA-P10*	*EA-P25*	*EA-P50*	*EA-P75*	*EA-P90*	*EA-P95*	*EA-P5*	*EA-P10*	*EA-P25*	*EA-P50*	*EA-P75*	*EA-P90*	*EA-P95*
*1*	2.800	3.140	3.480	4.000	4.350	4.870	5.200	49	50	52	54	57	59	60
*2*	3.220	3.260	3.960	4.600	5.010	5.600	5.700	49	50	54	57	59	62	64
*3*	3.640	3.850	4.555	4.920	5.550	6.100	6.300	50	53	58	60	63	64	65
*4*	3.790	4.000	5.250	6.445	7.130	7.910	8.200	56	57	62	64	66	69	71
*5*	4.750	5.100	5.700	6.600	7.180	8.100	8.900	58	59	63	65	67	70	70
*6*	5.400	5.500	6.570	7.240	7.960	8.400	8.700	62	62	64	68	70	71	71
*7*	5.800	5.800	5.900	7.150	8.000	10.000	10.000	63	66	66	69	70	74	74
*8*	6.300	6.300	7.475	8.000	8.450	9.900	9.900	66	66	68	72	73	76	76
*9*	6.400	6.500	7.300	7.820	8.850	9.050	9.170	67	67	68	69	73	75	75
*10*	6.500	7.500	8.000	8.600	9.060	9.980	10.150	67	70	72	74	76	78	79
*11*	6.600	6.950	8.005	8.700	9.320	10.500	11.300	70	70	71	73	74	78	78
*12*	3.450	7.700	8.000	9.800	10.400	10.900	11.000	68	72	73	75	78	79	82
*13–15*	7.000	7.000	7.400	8.980	10.000	10.300	12.400	72	73	74	76	82	86	87
*16–18*	9.000	9.000	9.200	10.500	10.900	11.500	11.500	73	73	78	80	82	86	89
*19–21*	9.300	9.600	10.000	11.200	12.400	13.100	14.000	79	80	81	83	85	87	89
*22–24*	9.500	9.700	10.500	12.000	13.300	14.000	14.000	80	82	83	86	90	91	91
*25–30*	8.000	8.000	11.100	13.500	14.400	14.600	14.600	80	80	86	90	95	95	96
*31–36*	10.900	11.000	12.200	14.000	15.000	16.800	17.100	89	90	92	94	98	99	100
*37–42*	11.500	11.500	12.700	14.000	16.100	17.800	17.800	88	88	93	96	100	101	101
*43–48*	12.500	12.700	13.000	14.900	17.000	18.700	20.500	95	97	97	100	104	107	108
*49–54*	11.800	13.900	14.500	16.000	18.500	19.500	21.600	97	99	101	106	110	111	114
*55–60*	13.000	15.000	15.000	17.000	18.600	19.000	19.100	103	104	105	107	110	111	114
*31–66*	14.950	14.950	16.800	20.650	21.750	22.700	22.700	108	108	110	114	117	123	123
*67–72*	14.500	14.500	16.000	20.400	22.400	25.400	25.400	101	101	113	114	116	121	121

**Table 3:** Percentiles for bodyweight and –height in females with esophageal atresia according to age (months) after multiple imputation. Germany, 2001–2021. Source: patient registry of KEKS e.V.

	*Weight (gr)*							*Height (cm)*						
	*EA-P5*	*EA-P10*	*EA-P25*	*EA-P50*	*EA-P75*	*EA-P90*	*EA-P95*	*EA-P5*	*EA-P10*	*EA-P25*	*EA-P50*	*EA-P75*	*EA-P90*	*EA-P95*
*Age (months)*														
*1*	2.840	3.050	3.300	3.750	4.270	4.610	5.050	43	43	50	53	56	59	62
*2*	3.500	3.790	4.070	4.410	5.000	5.420	5.710	51	52	55	58	60	61	64
*3*	3.690	3.910	4.320	5.200	5.400	6.030	6.450	52	53	57	60	63	65	65
*4*	4.320	4.480	5.000	5.310	5.830	7.330	7.800	58	58	59	61	63	65	66
*5*	4.420	4.540	5.800	6.915	7.500	8.500	9.000	58	58	62	66	68	70	71
*6*	5.600	6.100	6.300	6.925	7.435	7.680	7.860	61	61	62	65	67	67	70
*7*	6.980	6.980	7.000	7.000	7.360	7.600	7.600	62	66	66	66	69	70	70
*8*	5.500	5.500	6.160	7.900	9.050	9.700	9.700	62	62	63	66	71	72	72
*9*	5.620	6.060	6.925	7.500	8.490	8.500	9.200	64	64	67	72	72	73	74
*10*	6.810	6.970	7.310	7.560	7.900	8.750	8.800	65	65	67	69	71	74	77
*11*	6.070	6.800	7.200	8.450	8.990	9.500	10.300	67	68	70	71	74	76	76
*12*	7.500	7.500	7.790	9.750	10.500	11.000	11.000	71	71	71	75	80	86	86
*13–15*	7.700	7.835	8.000	9.385	10.000	10.350	10.500	65	65	72	74	79	83	83
*16–18*	6.770	6.770	8.000	9.060	11.500	12.000	12.000	72	72	75	80	83	87	87
*19–21*	8.000	8.000	9.600	10.200	11.550	11.800	12.000	76	76	79	83	84	85	86
*22–24*	9.000	9.200	11.000	12.100	13.300	14.500	14.500	79	79	81	86	89	93	93
*25–30*	8.000	9.200	10.000	12.100	13.400	14.000	14.100	80	81	84	88	90	93	97
*31–36*	8.950	8.970	11.500	13.100	15.100	17.000	18.000	79	82	88	95	97	102	103
*37–42*	10.700	10.800	11.700	14.700	15.500	17.300	19.000	88	89	91	96	100	103	110
*43–48*	10.500	10.600	13.300	15.000	16.900	17.450	19.500	87	87	97	100	105	108	110
*49–54*	13.600	13.800	14.850	17.150	18.650	20.000	20.900	96	101	104	106	109	111	112
*55–60*	11.000	11.000	14.200	18.500	19.600	22.000	22.000	94	94	106	112	113	116	116
*31–66*	17.500	17.500	17.500	19.200	20.000	21.000	21.000	112	112	113	116	116	120	120
*67–72*	14.100	14.100	16.150	18.000	22.100	25.000	25.000	98	98	105	115	118	121	121

### Impact of congenital heart disease

The information on presence or absence of congenital heart disease (CHD) was available for 276 patients (91.7%). At birth, there was no difference in gestational age between children with or without additional congenital heart disease (Table 4). Children with CHD had a lower z-score for birth weight and length (Table 4). During the first year of life, the presence of additional CHD has a substantially higher impact on body weight than on height. At the age of 13 to 24 months, CHD was associated with a higher median weight compared to EA patients without CHD, especially in females (Supplement 1a/ 1b). Due to sparse information in the older age groups of 13 to 24 months and more pronounced of25 to 72 months, estimates of the impact of CHD were characterized by wide confidence intervals (Supplement 1a/ 1b).

**Table 4:** Means and 95%-confidence intervals (95%-CI) of gestational age and biometric z-scores *according to Voigt [15] of esophageal atresia patients with and without additional congenital herat disease (CHD). Germany, 2001–2021. Source: patient registry of KEKS e.V.

	No CHD	CHD
	Mean (95%-CI)	Mean (95%-CI)
Gestational week	37.1 (36.6; 37.7)	37.1 (36.5; 37.8)
Z-score birthweight	−1.0 (−1.1; −0.8)	−1.1 (−1.3; −0.9)
Z-score length at birth	−0.4 (−0.8; −0.3)	−0.8 (−1.0; −0.5)

## Discussion

Our study provides percentiles for caregivers and health professionals specifically for children born with esophageal atresia, which have previously not been available. Compared to the general population, patients with EA are born with a lower birth weight and -length, and thereafter tend to follow a different, specific growth pattern. Furthermore, male infants and infants with additional congenital heart disease had a lower mean z-score for body weight at birth.

During the past decades, improvements in neonatal and surgical care, as well as early nutrition interventions have reduced the risk of malnutrition [2]. In our study, median weight for children with EA develops along the tenth centile of the general population. While the median height is close to normal during the first 6 months of life, growth decelerates thereafter in the sense that height crosses percentiles between the ages of 6 and 15 months and develops along the tenth centile of the general population until the age of 6 years. This finding corroborated the results of the French EA Registry, showing no catch-up growth during the first year of life, but a harmonious growth retardation in some patients at the age of one year [5]. Feeding problems seem to have an immediate impact in body weight, but a delayed impact on body height in patients with EA. Typically, more solid foods are introduced into an infant’s diet at the age of five to six months in order to promote age-appropriate development. In EA patients, esophageal strictures that had no impact on a formula diet, may become symptomatic and impact on adequate nutrition. They therefore should be identified and treated without delay in this important and consequential developmental phase. An additional adverse factor may be that parents typically hesitate to introduce solid foods. Therefore, children require close developmental, as well as weight and height surveillance during this vulnerable phase to detect imminent problems. Dysphagia can have many underlying causes in EA patients and is a common problem with a prevalence ranging from 21 to 84% of patients of all ages [2].

About one third of our cohort was born prematurely. This fact was taken into account by correcting the chronological age for the gestational age regarding all data on body weight and length during the first year of life. The influence of additional CHD was analysed by quartile regression and demonstrated an effect that should be taken into account, especially for older children with additional CHD while regarding our percentiles.

Long-time follow-up demographic data is scarce for patients born with EA. According to a recent study, EA patients still have a lower mean z-score for body weight (− 1.4) and height (− 0.7) in adolescence around the age of 15 [21]. One study reported that 75% of 13-year-old patients had a normal nutritional status, 16% were obese and 9% were malnourished [22]. Another study found a normal nutritional status in 68% of adolescents and young adults with EA at the age of 14 through 18 years [23]. Interestingly, in patients older than 18 years, there is a near-normal distribution of percentiles for body weight and –height [7]. Further studies in the adolescent and adult population are needed to determine at what age catch-up growth and weight gain occur.

### Strengths and limitations

This is the first study derived from a large national sample of patients born with esophageal atresia in Germany. Data quality is limited by the voluntary nature of data entry to the registry. While in some cases, information such as sex is missing, the overall data is qualitatively and quantitatively robust. As expected, registry entrances decrease with increasing age, leading to a larger mathematically-induced variation in the percentile curves (Figs. 2 and 3) in older children. We overcame these limitations by employing a multiple imputation approach for missing data.

It is also important to note that the growth data is cross-sectional for patients with esophageal atresia and not disease-specific. Co-morbidities may impact on growth, as well as the complexity of the individual patient’s case. Addressing the effects of different co-morbidities and other individual factors should be part of larger, future studies.

## Conclusion

Children with EA had a reduced bodyweight and -height compared to the general population. The age-based introduction of solid foods is a vulnerable phase, where occult problems, such as strictures, may become symptomatic and must be addressed without delay in order to ensure timely development and growth. Cardiac co-morbidities further compromise weight gain in these patients, implying that such patients should be under even closer surveillance.

## Supplementary Information

**Additional file 1: Supplement 1.** Median regression estimates and 95% confidence intervals of the influence of the parameter congenital heart disease on median weight (**a**) and height (**b**) of children with esophageal atresia in quartile regression according to age group. Germany, 2001–2021. Source: patient registry of KEKS e.V.

## Data Availability

The datasets analysed during the current study are not publicly available and are property of the patient organisation KEKS e.V. Selected data is available from the corresponding author (Tatjana.Koenig@unimedizin-mainz.de) on reasonable request.

## Abbreviations

CHD: Congenital heart disease
EA: Esophageal atresia
GER: Gastroesophageal reflux
95%-CI: 95%-Confidence Interval
