# Supplementary material for: Sex-specific percentiles for bodyweight and height in children born with esophageal atresia: a registry-based analysis 2001–2021
Source: BMC Pediatr. 2023 Jan 18;23:27. doi: 10.1186/s12887-023-03842-4 (PMC9847123; doi:10.1186/s12887-023-03842-4)

**Supplement 1** Median regression estimates and 95% confidence intervals of the influence of the parameter congenital heart disease on median weight (a.) and height (b.) of children with esophageal atresia in quartile regression according to age group. Germany, 2001-2021. Source: patient registry of KEKS e.V.


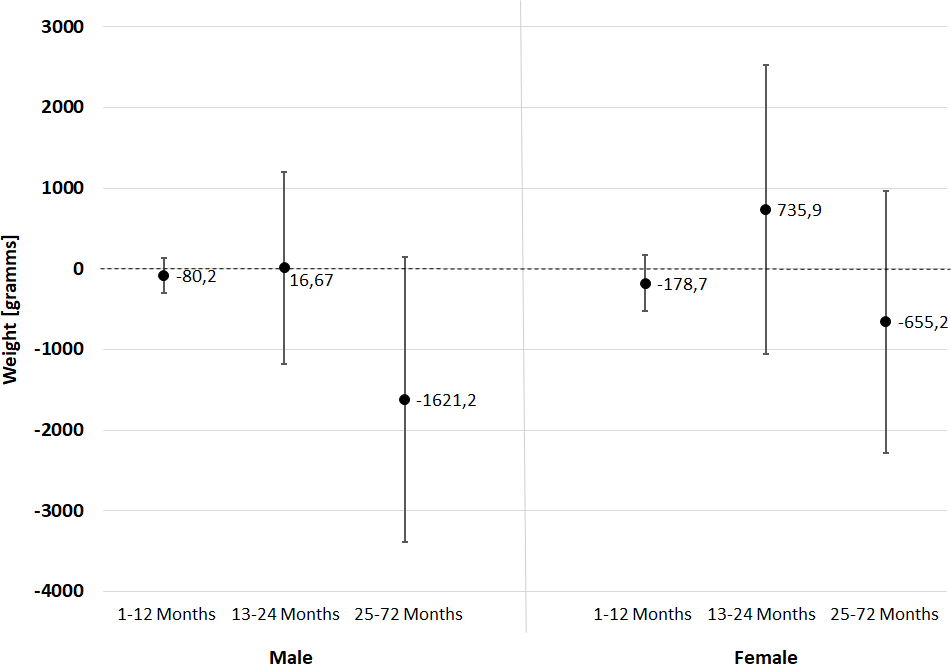


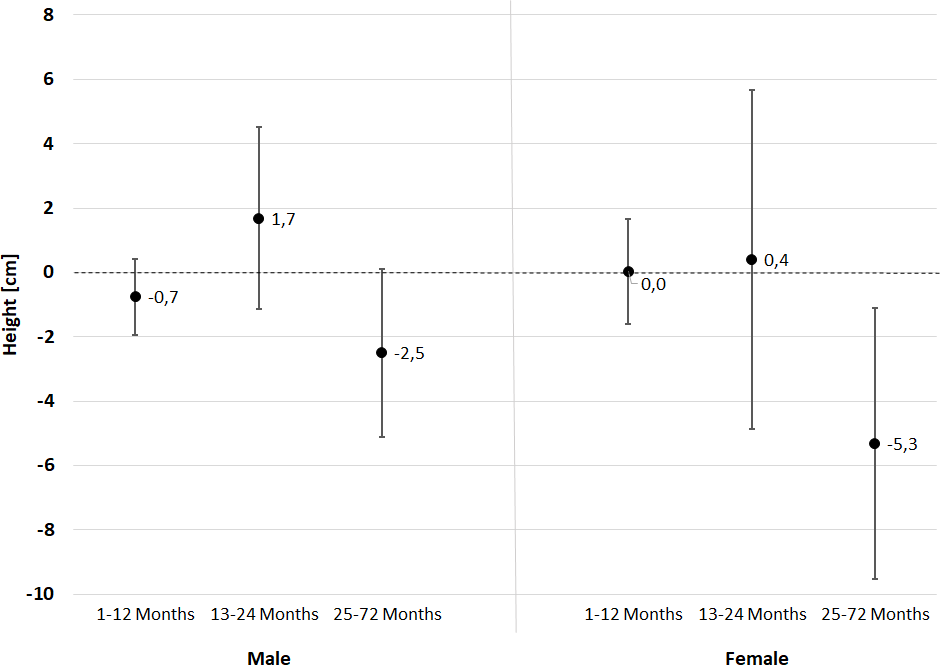

Supplement: Supplementary file 1 — Additional file 1: Supplement 1. Median regression estimates and 95% confidence intervals of the influence of the parameter congenital heart disease on median weight (a) and height (b) of children with esophageal atresia in quartile regression according to age group. Germany, 2001–2021. Source: patient registry of KEKS e.V. [file 12887_2023_3842_MOESM1_ESM.docx]
